# Microbiota-derived metabolic priming of CAR-T cell fitness: a translational framework for next-generation cell therapy

**DOI:** 10.3389/fimmu.2026.1953443

**Published:** 2026-09-22

**Authors:** Simone Melchiorri, Ignazio Castagliuolo

**Affiliations:** 1 Department of Medicine, University of Padua, Padova, Italy; 2 Department of Molecular Medicine, University of Padua, Padova, Italy

**Keywords:** CAR-T cell therapy, gut microbiota, immunometabolism, microbial metabolomics, microbiota-derived metabolites, short-chain fatty acids

## Abstract

Chimeric antigen receptor T-cell (CAR-T) therapy has transformed the treatment of hematologic malignancies, yet durable responses remain inconsistent and clinically significant toxicities persist. Emerging evidence identifies the gut microbiota as an extratumoral determinant of CAR-T efficacy, persistence, and toxicity. However, taxonomic signatures associated with clinical outcomes show limited reproducibility and are influenced by antibiotics, geography, diet, and methodological heterogeneity. Building on prior reviews addressing microbiome–CAR-T associations and CAR-T-cell immunometabolism separately, here we synthesize clinical, translational, and preclinical evidence into an integrated, metabolome-centered framework spanning the full CAR-T treatment and manufacturing trajectory. Broad-spectrum antibiotic exposure disrupts microbial metabolic output, depleting short-chain fatty acids, indole derivatives, and other bioactive metabolites, and has been associated with inferior clinical outcomes in several cohorts, although these associations are heterogeneous and may be confounded by baseline disease severity, systemic inflammation, and infection-related factors. Experimental studies further indicate that butyrate, valerate, inosine, indoles, and succinate modulate mitochondrial fitness, memory differentiation, epigenetic programming, cytotoxicity, and exhaustion through interconnected metabolic and receptor-mediated pathways. We propose that the gut microbiota acts as a distributed metabolic organ whose collective output shapes CAR-T-cell fitness and therapeutic response. This framework supports the development of metabolite-based biomarkers and therapeutic strategies, including microbiota-conscious antibiotic stewardship, ecosystem restoration, dietary or metabolite-based interventions, and *ex vivo* metabolic conditioning during CAR-T manufacturing. Shifting from descriptive microbiome profiling to functional metabolomics may improve both prediction and optimization of CAR-T therapy.

## 1 Introduction

Chimeric antigen receptor T-cell (CAR-T) therapy has transformed the treatment of hematologic malignancies, providing durable responses in patients with relapsed or refractory disease who often lack alternative therapeutic options (1, 2). Approved indications now include large B-cell lymphoma, B-cell acute lymphoblastic leukemia, mantle cell lymphoma, follicular lymphoma, and multiple myeloma, underscoring the growing clinical impact of this approach (1). However, therapeutic outcomes remain heterogeneous. Many patients fail to achieve durable remissions or develop severe toxicities, including cytokine release syndrome (CRS), immune effector cell-associated neurotoxicity syndrome (ICANS), and B-cell aplasia (3). In addition, resistance may result from limited CAR-T-cell persistence, progressive exhaustion, antigen escape, and an immunosuppressive tumor microenvironment (2).

The persistence of treatment failure despite continuous advances in CAR design and manufacturing suggests that host-related factors substantially influence therapeutic efficacy and toxicity (4). Among these, the gut microbiota has emerged as a potentially modifiable extratumoral determinant of CAR-T outcomes. Increasing evidence indicates that microbiota composition and function influence therapeutic response, persistence, resistance, and treatment-related toxicity, extending observations previously reported for immune checkpoint inhibitors and other forms of cancer immunotherapy (3, 5, 6).

The relevance of the gut microbiota to CAR-T-cell biology has been addressed in prior reviews focused on microbiome–CAR-T associations (1–3) and, separately, in reviews centered on CAR-T-cell immunometabolism without a specific focus on microbial contributions (7–11). Building on this foundation, the distinct contribution of the present review is not the introduction of a new concept, but the integration of microbiota-derived metabolic output with the full CAR-T treatment and manufacturing trajectory — spanning pre-infusion dysbiosis and antibiotic exposure, leukapheresis and *ex vivo* manufacturing, and post-infusion persistence and toxicity — within a single, metabolome-centered framework.

One of the strongest clinical observations supporting this concept comes from studies of antibiotic exposure. In CAR-T recipients, pre-treatment broad-spectrum antibiotics have been associated with shorter overall survival, increased ICANS, and a trend toward higher CRS. More broadly, antibiotic-induced dysbiosis, reduced microbial diversity, impaired barrier function, and expansion of pro-inflammatory microbial communities have been linked to diminished antitumor immunity and increased treatment-related toxicity across multiple immunotherapy settings (5, 6).

Despite these observations, most available studies remain largely associative and continue to focus on microbial taxonomy. Here, we argue that the relationship between the microbiota and CAR-T therapy is better understood through microbial metabolic function rather than taxonomic composition alone. We synthesize emerging clinical, translational, and preclinical evidence supporting microbiota-derived metabolites as the mechanistic link between the intestinal ecosystem and CAR-T-cell biology. By proposing a metabolome-centered framework, we aim to shift the field from descriptive microbial signatures toward functionally informed biomarkers and therapeutic strategies.

## 2 Microbiota-derived metabolites and CAR-T cell fitness

The gut microbiota modulates host immunity through bioactive metabolites, particularly short-chain fatty acids (SCFAs) and tryptophan-derived compounds, which regulate T-cell function through receptor-mediated and epigenetic mechanisms (7). Increasing evidence suggests that these pathways may also influence CAR-T-cell biology.

### 2.1 Immunometabolic determinants of CAR-T-cell function

CAR-T-cell efficacy depends not only on antigen recognition and CAR design but also on the immunometabolic state of the engineered T cell. Metabolic fitness is closely associated with persistence and resistance to exhaustion, with less differentiated, memory-like CAR-T-cell phenotypes displaying greater reliance on oxidative metabolism and being associated with superior persistence and antitumor activity compared with more differentiated effector phenotypes (8–11).

Mitochondrial fitness and epigenetic regulation are central components of these programs. Evidence from CAR-T studies supports an association between preserved mitochondrial fitness and improved persistence and functional capacity (10). Mechanistic studies in conventional T cells further demonstrate that mitochondrial dysfunction can promote the transition toward terminal exhaustion through metabolic and transcriptional reprogramming (12). Similarly, evidence from conventional T-cell biology indicates that nutrient-derived metabolites can influence chromatin accessibility and transcriptional programs governing memory and effector differentiation, although the extent to which these mechanisms operate identically in CAR-T cells remains incompletely defined (10, 12).

After infusion, CAR-T cells encounter a metabolically hostile tumor microenvironment characterized by nutrient deprivation, hypoxia, lactate accumulation, adenosine signaling, and altered amino acid availability (8–10). CAR-T-focused studies implicate these metabolic constraints in impaired cellular fitness and function, while mechanistic evidence from conventional tumor-infiltrating T cells demonstrates that tumor-driven nutrient competition can directly suppress T-cell metabolic and effector activity even in the presence of antigen recognition (13).

Within this framework, microbiota-derived metabolites emerge as plausible regulators of CAR-T-cell fitness. Evidence from conventional T-cell and broader immunological studies indicates that SCFAs generated by microbial fermentation can modulate T-cell differentiation, cytokine production, metabolic programming, and epigenetic regulation through receptor-mediated signaling and histone deacetylase (HDAC) inhibition (7, 14). Direct CAR-T evidence has more recently shown that butyrate treatment can promote memory-associated phenotypes, reduce exhaustion markers, and enhance antitumor activity in experimental CAR-T models (15).

Additional microbiota-derived metabolites may act through complementary regulatory pathways. Evidence from broader T-cell and microbiome immunology indicates that tryptophan-derived indoles can signal through the aryl hydrocarbon receptor (AhR), although direct evidence for this mechanism in CAR-T cells remains limited (7, 14). By contrast, inosine is supported by direct CAR-T evidence: higher serum levels have shown an exploratory association with improved progression-free survival in CAR-T recipients, while *in vitro* exposure increased CAR-T-cell cytotoxicity in an A2A receptor-dependent manner (16).

Collectively, these findings support a mechanistic framework in which microbiota-derived metabolites may influence CAR-T-cell biology through metabolic reprogramming, epigenetic regulation, and receptor-mediated signaling. However, the strength of evidence varies across these pathways, ranging from direct CAR-T studies to extrapolation from conventional T-cell and broader immunological models, providing the conceptual basis for the clinical observations discussed in subsequent sections.

## 3 Antibiotics and the microbiota: a modifiable determinant of CAR-T outcomes

### 3.1 Prevalence and clinical relevance of antibiotic exposure

Antibiotic exposure is common among patients receiving CAR-T therapy because of prior treatment, immunosuppression, infectious complications, and empiric management of neutropenic fever. In the multicenter cohort reported by Smith et al., 228 patients with non-Hodgkin lymphoma (n = 137) or acute lymphoblastic leukemia (n = 91) receiving anti-CD19 CAR-T therapy were evaluated for antibiotic exposure during the 4 weeks preceding infusion; approximately 60% received at least one antibiotic. The most frequently used agents included trimethoprim-sulfamethoxazole, intravenous vancomycin, piperacillin-tazobactam, levofloxacin, cefepime, ciprofloxacin, and meropenem. Particular attention was given to the broad-spectrum agents piperacillin-tazobactam, imipenem-cilastatin, and meropenem (P-I-M), which have substantial activity against obligate anaerobes; 47 of 228 patients (20.6%) were exposed to at least one of these agents within the 4-week pre-infusion window (17).

A subsequent study by Prasad et al. evaluated 422 recipients of anti-CD19 CAR-T therapy for relapsed/refractory lymphoma and used a 6-week pre-infusion exposure window. Overall, 171 patients (40.5%) received antibiotics, while 64 (15.2%) were exposed to antibiotics with substantial anti-anaerobic activity, including piperacillin-tazobactam, carbapenems, metronidazole, and clindamycin. In this cohort, antibiotic-exposed patients also had higher tumor burden, greater systemic inflammation, and more extensive prior therapy, emphasizing that antibiotic exposure may partly identify patients with greater baseline clinical complexity rather than representing an isolated causal determinant of outcome (18).

Thus, antibiotic exposure before CAR-T infusion is frequent, but its prevalence and biological relevance depend on the exposure window, antibiotic spectrum, and patient population. In particular, broad-spectrum regimens with substantial anti-anaerobic activity appear to produce the greatest microbiome disruption and have therefore been the principal focus of clinical outcome studies. However, these observational associations must be interpreted in the context of substantial confounding by disease burden, systemic inflammation, infection, and prior treatment intensity.

### 3.2 Clinical evidence and relationship with outcomes

Clinical studies have associated pre-infusion antibiotic exposure with inferior CAR-T outcomes, although the strength of this association varies according to antibiotic spectrum, exposure window, and baseline clinical risk. In Smith et al., 228 patients with NHL or ALL receiving anti-CD19 CAR-T therapy were evaluated over the 4 weeks before infusion. Exposure to piperacillin-tazobactam, imipenem-cilastatin, or meropenem (P-I-M; n = 47) was associated with shorter OS and PFS and increased ICANS; importantly, P-I-M exposure remained independently associated with shorter OS after multivariable adjustment (HR 2.54, 95% CI 1.62–3.97, P <.001) (17).

Prasad et al. analyzed 422 anti-CD19 CAR-T recipients using a 6-week pre-infusion window. Antibiotic exposure occurred in 171 patients (40.5%) and was associated with inferior PFS, OS, and higher relapse incidence; in the LBCL subgroup, it remained independently associated with poorer PFS (HR 1.50, P = .013). However, exposed patients also had greater tumor burden, systemic inflammation, and more prior therapy, highlighting substantial confounding by indication (18).

Stein-Thoeringer et al. studied 172 patients with B-cell lymphoma across German and US centers using a 3-week pre-infusion window. “High-risk” broad-spectrum antibiotics—meropenem, cefepime, ceftazidime, and piperacillin-tazobactam—were associated with increased progression and reduced PFS and OS. After adjustment for age, ECOG, country, LDH, CRP, bulky disease, and prior therapies, the association persisted for PFS (HR 2.19) but not OS, again indicating that baseline disease severity accounts for part of the observed effect (19).

More recently, Bullegas et al. retrospectively evaluated 140 patients with r/r DLBCL receiving CD19-directed CAR-T therapy, of whom 67 had received infection-related antibiotics within the preceding 6 months. Exposure encompassed multiple antibiotic classes, including cephalosporins, nitroimidazoles, penicillins, glycopeptides, and carbapenems, thus including agents with substantial anti-anaerobic activity. Antibiotic exposure was associated with inferior PFS and OS and remained independently associated with both outcomes after multivariable adjustment for age, comorbidity burden, remission status, and CAR-T product (PFS: HR 1.67, 95% CI 1.07–2.60, P = .023; OS: HR 2.14, 95% CI 1.29–3.55, P = .003). Multiple antibiotic courses and shorter intervals between the last exposure and CAR-T infusion were also associated with poorer outcomes; however, the retrospective design and potential confounding by infection and baseline clinical status preclude causal inference (20).

Post-infusion antibiotic exposure represents a clinically relevant but less well-characterized setting. Broad-spectrum antibiotics are frequently required after CAR-T infusion for febrile neutropenia and infectious complications. In longitudinal analyses, high-risk antibiotic exposure was associated with reduced microbial diversity across longitudinally collected samples, while studies of idecabtagene vicleucel recipients demonstrated a significant decline in gut microbial diversity after infusion and post-treatment expansion of facultative pathobionts (19, 21). However, these post-infusion microbiome changes cannot be attributed specifically to antibiotic exposure. Importantly, the available clinical studies have primarily evaluated survival associations using pre-infusion antibiotic exposure windows, and the independent effect of post-infusion antibiotics on CAR-T efficacy therefore remains poorly defined. This question is particularly difficult to address because post-infusion antibiotic use is strongly confounded by febrile neutropenia, infection, hospitalization, and treatment-related toxicity. Prospective studies incorporating time-dependent antibiotic exposure and longitudinal microbiome profiling are needed to disentangle these effects.

Preclinical data also indicate that antibiotic effects are context dependent. Kuczma et al. found that broad-spectrum antibiotics impaired cyclophosphamide-induced and CD4+ T-cell-mediated antitumor responses, but did not reduce the efficacy of CD19-directed CAR-T cells in a murine B-cell lymphoma model (22). Overall, current evidence supports an association between selected broad-spectrum, microbiome-disruptive antibiotics and inferior CAR-T outcomes in several cohorts, but does not establish a uniform or causal effect.

### 3.3 Confounding and causal plausibility

Interpretation of clinical associations remains complicated by confounding by indication, because antibiotic-treated patients often have greater tumor burden, systemic inflammation, infectious complications, poorer performance status, and more extensive prior therapy. This was particularly evident in the Stein-Thoeringer cohort, where adjustment for baseline clinical variables attenuated the association with OS, although an independent association with PFS persisted (19). Conversely, Smith, Prasad, and Bullegas reported that antibiotic exposure remained independently associated with selected adverse outcomes after multivariable adjustment (17, 18, 20).

Accordingly, the available clinical data support association rather than causation. Stronger causal plausibility comes from preclinical experiments: Prasad et al. showed that meropenem-induced dysbiosis in an immunocompetent lymphoma model altered microbial metabolic output and reduced anti-CD19 CAR-T efficacy (18). However, the absence of a detrimental effect in some other CAR-T models further indicates that antibiotic effects are context dependent rather than uniformly causal (22).

### 3.4 From taxonomic damage to metabolic collapse

A major conceptual advance in the field has been the shift from descriptive taxonomic analyses toward functional characterization of microbiome disruption. Antibiotics with substantial anaerobic activity have been associated with depletion of key microbial metabolites, including SCFAs such as butyrate, propionate, and valerate, as well as indole and cresol derivatives, ursodeoxycholate, and trimethylamine N-oxide, together with loss of fermentative bacterial communities (18). Among circulating metabolites associated with improved PFS were trimethylamine N-oxide (TMAO), indoxyl sulfate, and cresyl sulfate. These compounds should be interpreted as microbiota-associated or microbial–host co-metabolites rather than exclusively microbiota-derived effectors, and their association with favorable outcomes does not establish a direct beneficial effect on CAR-T cells.

SCFAs remain particularly relevant because they provide a plausible functional link between microbiome activity and T-cell fitness. Butyrate and related SCFAs can modulate T-cell metabolism, differentiation, and epigenetic programs (7), while direct CAR-T studies have shown that butyrate exposure can enhance memory-associated phenotypes and cytotoxic function (23). These metabolic alterations have also been reproduced in preclinical models of antibiotic-induced dysbiosis, supporting a mechanistic link between microbiome disruption and impaired CAR-T efficacy (18). Overall, antibiotic-induced dysbiosis may affect CAR-T therapy not only through taxonomic depletion but also through loss of microbial metabolic function, although the extent to which these metabolic changes directly mediate clinical outcomes remains incompletely established. The proposed framework linking preserved microbiota or antibiotic-induced dysbiosis to microbial metabolic output, CAR-T-cell fitness, and clinical outcomes is summarized in **Figure 1**.

**Figure 1:**
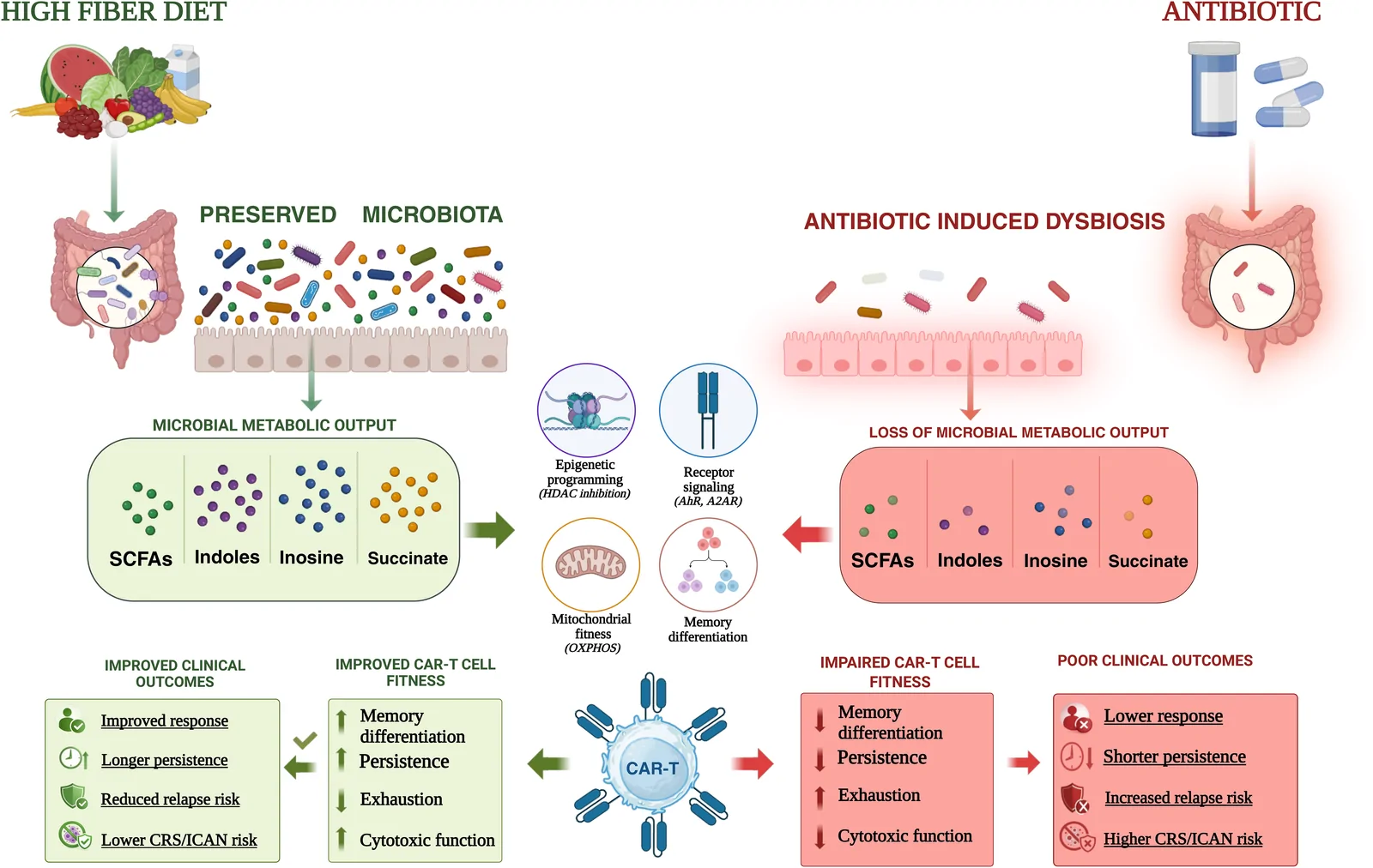
Microbiota–metabolome influences on CAR-T-cell fitness and clinical outcomes. High-fiber diet and antibiotic exposure may differentially shape gut microbiota, microbial metabolic output, CAR-T-cell fitness, and treatment outcomes. SCFAs, short-chain fatty acids; CRS, cytokine release syndrome; ICANS, immune effector cell-associated neurotoxicity syndrome.

### 3.5 Clinical implications: toward selective antibiotic stewardship

Current evidence does not support withholding antibiotics when clinically indicated but strongly argues for microbiome-conscious antibiotic stewardship. Whenever feasible, unnecessary exposure to broad-spectrum agents with extensive anaerobic coverage should be minimized and early de-escalation encouraged to preserve microbiome function (18, 24). Importantly, antibiotic effects are not universally detrimental. In preclinical models, oral vancomycin enhanced CAR-T efficacy through microbiota remodeling and improved antigen cross-presentation (25), while some prospective studies have not confirmed significant associations between prior high-risk antibiotic exposure and clinical outcomes (26). These findings highlight the complexity of host–microbiota interactions and reinforce the need for prospective mechanistic studies.

Overall, current evidence identifies antibiotic-induced disruption of the microbiota–metabolome axis as one of the most compelling and clinically actionable host determinants of CAR-T efficacy and toxicity, while emphasizing the need for prospective mechanistic validation.

## 4 Predictive microbial signatures of CAR-T response: from taxonomic correlations to the limits of composition

### 4.1 Baseline microbiota status: a dysbiotic ecosystem

At the time of CAR-T infusion, the gut microbiota rarely resembles that of a healthy individual. Prior chemotherapy, infections, and antibiotic exposure frequently lead to depletion of commensal anaerobes, enrichment of pathobionts, and impaired ecosystem function. Accordingly, patients consistently exhibit reduced microbial diversity, taxonomic dominance, and expansion of *Enterobacteriaceae* compared with healthy controls (16, 17, 26). Consequently, microbiota signatures associated with CAR-T outcomes should be interpreted within an already disrupted ecosystem rather than against the background of a healthy microbiota.

### 4.2 Predictive signatures of response and toxicity

Despite substantial cohort heterogeneity, several CAR-T studies have identified associations between favorable clinical outcomes and enrichment of bacterial taxa with immunomodulatory or SCFA-producing potential. In anti-CD19 CAR-T recipients, complete response has been associated with higher abundance of Clostridia, including *Ruminococcus* and *Faecalibacterium*, while other cohorts have reported associations involving *Faecalibacterium*, *Roseburia*, *Ruminococcus*, *Bifidobacterium*, and additional SCFA-producing taxa (17, 23, 27). However, the specific taxonomic signatures are not fully consistent across cohorts. Conversely, dysbiotic configurations characterized by taxonomic dominance or enrichment of facultative pathobionts have been associated with adverse CAR-T outcomes, with *Enterococcus* in particular linked to greater treatment-related toxicity in some cohorts (17, 21, 23).

Notably, some taxa associated with treatment response were also linked to toxicity-related patterns, indicating that microbiome associations with efficacy and safety are not necessarily concordant. For example, *Bifidobacterium* was enriched in complete responders in the multiple-myeloma cohort but was also associated with more severe CRS, and these associations were not consistently reproduced in the independent validation cohort. Such context-dependent findings underscore the limited reproducibility and interpretability of genus-level associations and support the need for functional rather than purely taxonomic analyses (27).

Human observational evidence currently spans several CAR-T products and hematologic malignancies, although the depth of microbiome characterization remains uneven across indications. Most available data derive from patients receiving CD19-directed CAR-T therapy for B-cell malignancies, including non-Hodgkin lymphoma, large B-cell lymphoma, diffuse large B-cell lymphoma, and acute lymphoblastic leukemia, in whom antibiotic exposure, baseline microbial composition, microbial diversity, and selected metabolite profiles have been associated with response, progression-free survival, overall survival, and treatment-related toxicity (16–20, 23, 27). In multiple myeloma, emerging observational evidence from BCMA-directed CAR-T therapy, including idecabtagene vicleucel, has documented longitudinal microbiome disruption and post-treatment changes in microbial diversity and composition (21). Across these studies, however, differences in CAR construct, underlying malignancy, prior treatment exposure, antibiotic use, geographic setting, sampling time point, and microbiome-analysis platform substantially limit direct comparison between cohorts. Thus, while human observational studies support a relationship between the microbiota–metabolome axis and CAR-T outcomes across more than one product and disease context, product- and malignancy-specific signatures remain insufficiently validated for clinical use.

Microbiota disruption may also contribute to CAR-T-related toxicity. Clinical CAR-T studies have associated dysbiosis and broad-spectrum antibiotic exposure with increased CRS and/or ICANS, although these relationships remain observational (1, 17, 28, 29). Mechanistically, studies outside the CAR-T setting suggest that impaired intestinal barrier integrity and microbial translocation can increase systemic exposure to microbial products such as lipopolysaccharide, whereas CAR-T-specific studies establish monocytes and macrophages as major mediators of CRS and neuroinflammatory toxicity (30–32). Whether microbiota disruption directly amplifies these myeloid inflammatory pathways in CAR-T recipients has not yet been demonstrated. Clinical and translational evidence linking the microbiota–metabolome axis to CAR-T outcomes is summarized in **Table 1**.

**Table 1:** Clinical and translational evidence linking the microbiota–metabolome axis to CAR-T outcomes.

Study (year)	Population	Main findings	Evidence type	Functional signal
Smith et al., *Nat Med* (17)	Lymphoma/ALL (anti-CD19)	↑ *Clostridia* in responders; antibiotics → ↑ mortality (HR 1.71, p = 0.011); ↑ ICANS (p = 0.002)	Multicenter observational	SCFA-producing taxa
Hu et al., *Nat Commun* (27)	Hematological malignancies	Microbial signatures (*Faecalibacterium, Bifidobacterium*) correlate with response; AUROC 0.85; microbiota modulates CRS	Observational	
Stein-Thoeringer et al., *Nat Med* (19)	Lymphoma (anti-CD19)	Non-dysbiotic microbiota → better response; antibiotics → ↓ PFS (independent effect in multivariate analysis)	Observational	
Prasad et al., *Blood* (18)	Lymphoma/other	Broad-spectrum antibiotics → ↓ SCFAs (butyrate, valerate), ↓ indoles, ↓ PFS; valerate = prognostic biomarker; effect replicated in murine models	Clinical + preclinical	SCFA depletion
Marcos-Kovandzic et al., *Cancer Discov* (33)	Murine models + clinical data	Baseline *Akkermansia muciniphila* positivity was associated with higher 6-month ORR and longer PFS; in a syngeneic murine model, Akkermansia massiliensis p2261 enhanced CD19 CAR-T activity partly via indole/AhR signaling, whereas IPA/indoles alone did not reproduce the full effect.	Translational	Indole/AhR
García-Vicente et al., *Clin Cancer Res* (23)	Non-Hodgkin lymphoma	↑ SCFA-producers in responders; butyrate *ex vivo* → ↑ CD8+ memory phenotype, ↑ CAR-T cytotoxicity	Observational + preclinical	Butyrate
Yoon et al., *Blood Adv* (16)	r/r DLBCL (anti-CD19)	↑ Serum inosine → improved PFS; submedian inosine (↓) independently predicts worse PFS (HR 2.78, p=0.024); AUROC 0.88 with inosine biosynthesis pathway; inosine ↑ CAR-T cytotoxicity *in vitro* (A2AR-dependent)	Observational + *in vitro*	Inosine

↑ increase/higher; ↓ decrease/lower. HR, hazard ratio; PFS, progression-free survival; ORR, overall response rate; AUROC, area under the receiver operating characteristic curve; CRS, cytokine release syndrome; ICANS, immune effector cell-associated neurotoxicity syndrome; SCFA, short-chain fatty acid; AhR, aryl hydrocarbon receptor; A2AR, adenosine A2A receptor.

### 4.3 Limits of taxonomic signatures and functional interpretation

The principal limitation of current microbiome studies is not the absence of taxonomic associations, but their poor reproducibility across independent cohorts, likely reflecting geographic, dietary, methodological, and clinical heterogeneity (16, 21). Moreover, genus-level analyses may obscure species- and strain-specific effects, as illustrated by conflicting findings involving Bifidobacterium and other taxa (26).

More importantly, taxonomy represents only a surrogate of biological function. Taxa associated with favorable outcomes largely share the ability to generate immunomodulatory metabolites, whereas expansion of pathobionts frequently reflects the loss of these metabolic functions. Accordingly, microbiota-derived metabolites provide a mechanistic framework capable of explaining CAR-T outcomes more consistently than taxonomic composition alone (18).

Taken together, these observations suggest that microbiome function is more informative than microbiome composition. Future biomarker strategies should therefore prioritize microbial metabolic output and host–microbiome interactions over taxonomic signatures alone, providing the conceptual foundation for the metabolome-centered framework discussed in the following sections. In other words, taxonomy identifies who is present, whereas metabolomics reveals what the microbial ecosystem is actually doing.

## 5 The microbial metabolome as a central functional level

### 5.1 Limits of taxonomy and function–composition decoupling

Taxa associated with favorable CAR-T outcomes, including Ruminococcus, Faecalibacterium, Prevotella, and Roseburia, converge functionally rather than phylogenetically, sharing the capacity to produce immunomodulatory metabolites. While taxonomy provides insight into metabolic potential, it cannot fully capture microbiome function: phylogenetically distinct microorganisms may generate similar metabolite profiles, whereas closely related organisms can produce markedly different metabolic outputs. Consequently, the biological impact of the microbiota is determined less by the presence of specific organisms than by the collective metabolic activity of the microbial ecosystem, a concept supported by studies linking antibiotic-induced metabolite depletion to inferior CAR-T outcomes (18). Thus, microbial composition defines the metabolic potential of the ecosystem, whereas the metabolome reflects its actual functional activity under a given physiological condition.

### 5.2 The metabolome as a clinical predictor

Consistent with this concept, several studies have identified fecal and circulating metabolites associated with CAR-T efficacy. These measurements should not be interpreted interchangeably, because stool concentrations primarily reflect intestinal metabolic output, whereas circulating concentrations integrate microbial production, host metabolism, absorption, and systemic clearance. Reduced SCFA concentrations, particularly butyrate, propionate, and valerate, have been associated with inferior outcomes, whereas higher valerate levels have been associated with improved progression-free survival (18, 23). Likewise, preservation of microbial indole pathways and higher serum inosine concentrations have been associated with favorable outcomes across independent cohorts (16, 21, 33).

### 5.3 The metabolome as a functional readout of a metabolic network

Together, these observations suggest that microbiome function may be captured more effectively through metabolomics than through sequencing alone. Targeted metabolite panels including butyrate, valerate, and inosine could provide a quantitative assessment of microbiome-associated metabolic function at the time of CAR-T infusion and may prove more clinically translatable than taxonomic profiling alone.

Importantly, identifying individual metabolites as biomarkers does not imply that single compounds can reproduce the effects of a complex microbiota. Experimental studies indicate that isolated metabolites often fail to recapitulate the coordinated biological activity of microbial communities (33). Consistently, responders exhibit coordinated alterations across multiple metabolite classes, suggesting that unfavorable outcomes reflect broader metabolic dysfunction rather than deficiency of a single metabolite.

This framework explains the limited reproducibility of taxonomic signatures across independent cohorts and identifies metabolite-centered approaches as a more biologically informative and clinically actionable strategy for biomarker discovery and therapeutic intervention. In this framework, taxonomy defines the microbial repertoire, whereas the metabolome represents the biologically active layer directly interacting with the host immune system.

### 5.4 Therapeutic implications

The metabolome may represent not only a source of biomarkers but also a potential therapeutic target. Direct CAR-T studies indicate that *ex vivo* exposure to SCFAs can modulate CAR-T-cell metabolism, providing a strategy that could reduce dependence on interindividual variability in host microbiota composition. In experimental CAR-T models, butyrate promotes memory-associated phenotypes and reduces senescence-associated features, whereas SCFAs more broadly enhance CAR-T metabolic fitness and tumor-killing capacity (18, 23). These translational opportunities are discussed further in the section “*Ex vivo* Metabolic Conditioning of CAR-T Cells”.

### 5.5 Conceptual synthesis

Collectively, current evidence suggests that the predictive value of the microbiota resides less in taxonomic composition than in metabolic function. Taxonomic signatures remain informative because they reflect underlying biochemical capabilities, but metabolites represent a more proximal biological signal linking the microbiota to host immunity and CAR-T-cell biology. The metabolome therefore emerges as a promising framework for both biomarker development and therapeutic intervention.

## 6 Microbial metabolites as candidate functional mediators of CAR-T efficacy

### 6.1 From association to causality

Microbiota-derived metabolites should be viewed not only as biomarkers of CAR-T outcomes but also as candidate functional mediators of CAR-T-cell biology. Emerging evidence indicates that specific metabolites directly influence CAR-T-cell metabolism, differentiation, persistence, and effector function through defined molecular pathways. Accordingly, the central question is no longer simply which microbial taxa are present, but whether microbiota-derived metabolites causally regulate therapeutic response.

Importantly, the metabolites discussed below should not be considered uniformly or exclusively microbiota-derived. Their abundance may reflect microbial production, host metabolism, dietary exposure, tumor metabolism, or combined microbial–host biotransformation. Moreover, fecal, circulating, and intratumoral measurements represent biologically distinct compartments and should not be interpreted interchangeably. Functional effects may further vary according to concentration, cellular and receptor context, and disease state. Accordingly, metabolite origin and measurement compartment should be considered when interpreting their associations with CAR-T outcomes and their potential biological effects.

The strength of evidence varies substantially across metabolite classes, ranging from clinical associations to direct mechanistic studies in CAR-T models. Accordingly, the following sections examine candidate metabolites according to both their mechanism of action and the level of experimental evidence supporting their role in CAR-T efficacy (**Table 2**). The principal biological origins, measurement compartments, and context-dependent interpretive considerations of the metabolites discussed below are summarized in **Table 3**. Detailed experimental models, CAR targets, exposure conditions, doses, principal endpoints, dose- or context-dependent effects, and translational evidence for the major metabolites discussed below are summarized in **Supplementary Table S1**.

**Table 2:** Evidence-source classification for microbiota- and metabolite-related mechanisms relevant to CAR-T therapy.

Evidence source	Definition	Examples	Interpretation
Direct clinical CAR-T evidence	Associations measured directly in patients receiving CAR-T therapy.	Antibiotic exposure and gut microbial signatures associated with efficacy and/or toxicity; circulating or fecal microbiota-associated metabolites, including SCFAs, succinate, and inosine, associated with CAR-T response, persistence, survival, or toxicity (16–19, 21, 23, 27, 34).	CAR-T-specific human evidence; predominantly observational and associative, with some metabolite findings exploratory or cohort-dependent.
Direct mechanistic CAR-T evidence	Experimental effects demonstrated directly in CAR-T cells or CAR-T animal models.	Butyrate, succinate, inosine, and kynurenine/AhR modulation of CAR-T phenotype or function (15, 16, 23, 34, 36).	Supports direct biological effects on CAR-T cells.
CAR-T microbiota-manipulation evidence	Experimental alteration of the microbiota followed by assessment of CAR-T efficacy	Antibiotic-induced dysbiosis, *Akkermansia*-dependent effects, and vancomycin-mediated microbiota remodeling (18, 25, 33).	Supports a causal microbiota–CAR-T relationship.
Indirect T-cell/immunology evidence	Mechanisms demonstrated in conventional T cells or broader immune models.	SCFA–HDAC signaling, metabolic/epigenetic programming, AhR signaling, methionine restriction, and lactate-mediated suppression (7, 12–14, 38, 42).	Mechanistically relevant but not direct CAR-T evidence.
Other immunotherapy/tumor-biology evidence	Evidence derived from checkpoint blockade or general tumor-immunity models.	Microbiota-dependent modulation of PD-L1, IDO1/kynurenine signaling, TME remodeling, and antitumor immunity (6, 30, 46).	Cross-platform extrapolation requiring CAR-T validation.
Translational/hypothetical evidence	Plausible interventions not yet adequately validated in CAR-T recipients.	FMT, dietary modulation, microbiome-sparing approaches, metabolite-based interventions, and *ex vivo* metabolic conditioning (6, 18, 23, 24, 49, 53).	Investigational; clinical relevance remains to be established.

CAR-T, chimeric antigen receptor T-cell; SCFA, short-chain fatty acid; HDAC, histone deacetylase; AhR, aryl hydrocarbon receptor; IDO1, indoleamine 2,3-dioxygenase 1; TME, tumor microenvironment; FMT, fecal microbiota transplantation.

**Table 3:** Biological origin, measurement compartment, and interpretative context of metabolites relevant to CAR-T therapy.

Metabolite	Biological origin	Relevant compartment	Key interpretative caveat
Butyrate	Predominantly microbial	Stool; experimental CAR-T exposure	Effects are concentration- and context-dependent
Valerate	Predominantly microbial	Stool; experimental CAR-T exposure	Clinical associations and direct functional effects should be distinguished
TMAO	Microbial–host co-metabolite	Circulation	Association does not imply direct CAR-T benefit
Indoxyl sulfate	Microbial–host co-metabolite	Circulation	Requires microbial precursor formation and host biotransformation
Cresyl sulfate	Microbial–host co-metabolite	Circulation	Clinical association does not establish causality
Succinate	Microbial and host-derived	Stool; experimental CAR-T models	Source and effects are context-dependent
Indoles	Predominantly microbial	Gut/metabolic pathways; experimental models	AhR effects are ligand- and context-dependent
Kynurenine	Predominantly host/tumor-derived	Tumor/metabolic context	Should not be considered a microbiota-derived indole
Inosine	Microbial and host-derived	Serum; microbial pathways	Serum levels cannot be attributed exclusively to microbial production
Lactate	Predominantly host/tumor-derived	Tumor microenvironment	Effects depend on concentration, acidity, and hypoxia

TMAO, trimethylamine N-oxide; CAR-T, chimeric antigen receptor T-cell; AhR, aryl hydrocarbon receptor.

The relative maturity of the evidence supporting the principal microbiota–metabolite pathways discussed in this review is summarized in **Figure 2**, distinguishing established CAR-T biological principles from emerging direct evidence and hypothetical mechanisms that remain largely extrapolated or unvalidated in CAR-T-specific settings.

**Figure 2:**
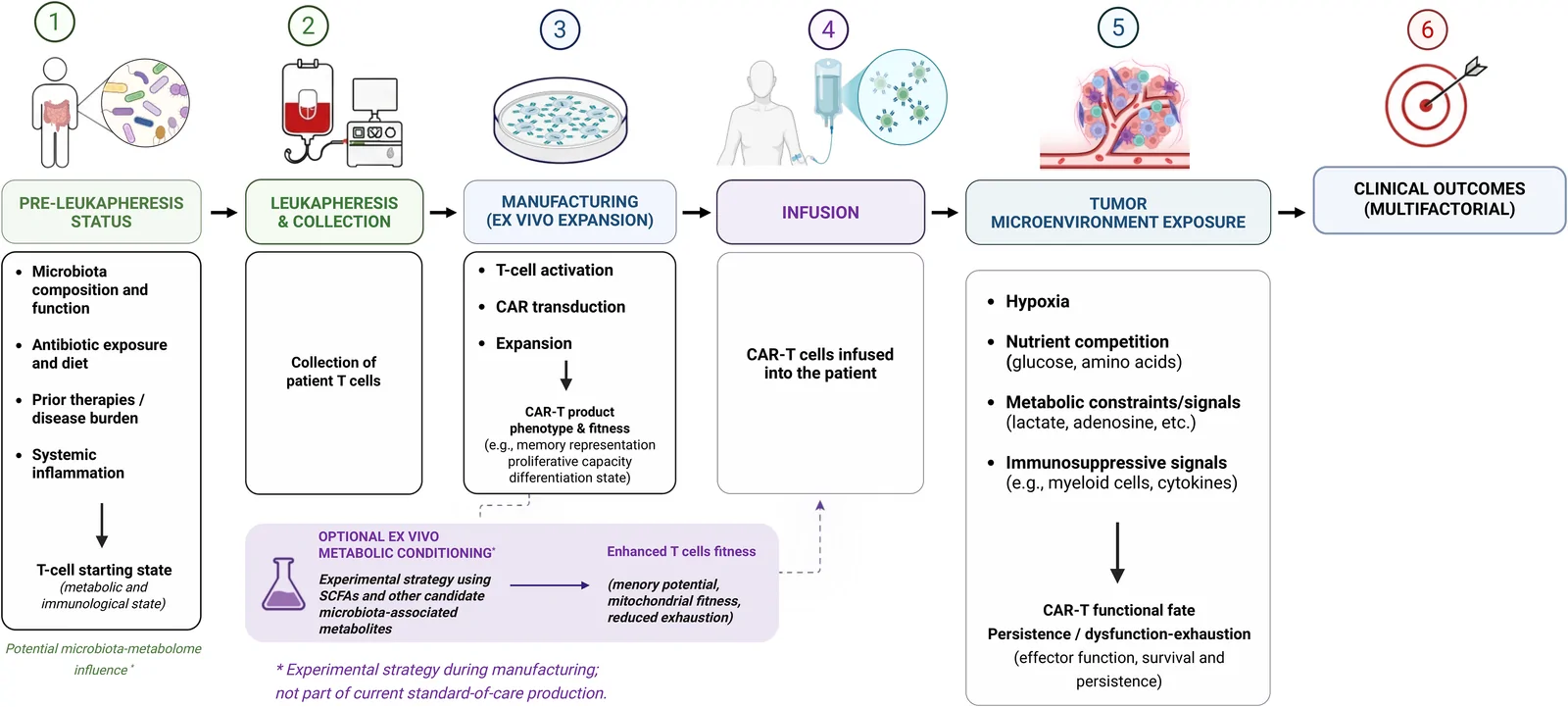
Microbiota–metabolome influences across the CAR-T-cell therapeutic trajectory. Schematic overview of pre-leukapheresis status, T-cell collection, *ex vivo* manufacturing, infusion, tumor microenvironment exposure, and clinical outcomes, highlighting potential metabolic and host-related influences at each stage.

### 6.2 Energetic metabolites: succinate, butyrate, and valerate

Several microbiota-associated metabolites have been implicated in pathways related to cellular metabolism, mitochondrial function, and epigenetic programming, although the level of direct CAR-T evidence varies substantially among individual metabolites. In selected CAR-T models, these metabolic pathways may influence memory differentiation, persistence, and functional fitness.

Mechanistically, butyrate, valerate have each been linked, at least in part, to inhibition of class I histone deacetylases (HDACs) — pentanoate acting as a specific class I HDAC inhibitor — a shared epigenetic mechanism believed to promote the histone hyperacetylation associated with memory-related transcriptional programs in T cells (7, 14). Succinate, by contrast, is thought to act primarily through mitochondrial/oxidative-phosphorylation pathways rather than HDAC inhibition. Whether excessive HDAC blockade at supraphysiological concentrations could impair rather than enhance CAR-T-cell proliferation and activation — a narrow therapeutic window well documented for HDAC inhibitors in other T-cell contexts — has not yet been directly tested for these metabolites in CAR-T-specific models.

Succinate is supported by direct CAR-T evidence across clinical, *in vitro*, and murine settings (34). In patients with relapsed/refractory multiple myeloma receiving BCMA-directed CAR-T therapy (ARI0002h, a 4-1BB-based construct), higher pre-infusion stool succinate levels, measured by NMR, correlated with CAR-T-cell persistence at day 100 post-infusion in the evaluable subset (n = 18), as assessed by vector copy number per genome (Spearman R = 0.444, P = 0.044). *In vitro*, exogenous succinate supplementation (20 mmol/L during the final 3 days of CAR-T expansion) increased the proportion of CD4^+^ central-memory cells, decreased CD4^+^ effector-memory cells, and significantly enhanced spare respiratory capacity, consistent with a memory-like metabolic profile; no significant change in cytotoxicity against U266 myeloma target cells or in exhaustion markers was observed. In a murine ARP-1 xenograft model treated with ARI0002h CAR-T cells, mice fed a fructooligosaccharide (FOS)-supplemented diet, known to increase intestinal succinate levels, showed enhanced T-cell persistence and improved tumor control, although overall survival was unchanged. Notably, these findings contrast with an independent report in which 5 mmol/L succinate for 72 hours impaired CD4^+^ T-cell cytotoxic capacity, an apparent discrepancy attributed by the authors to differences in succinate concentration and exposure duration. These observations suggest that succinate effects on CAR-T-cell function are exposure- and context-dependent rather than uniformly beneficial.

Among SCFAs, butyrate is currently one of the most extensively investigated metabolites in the CAR-T setting, with direct evidence spanning solid-tumor and hematologic CAR-T platforms, both murine and human, and *in vitro* through *in vivo* models.

In a mesothelin (MSLN)-directed CAR-T model developed for ovarian and breast cancer (human CAR-T cells tested against HCC1806, OVCAR3, and SKOV3 target lines, with confirmatory xenografts in C-NKG mice), sodium butyrate (2 mM, 2-day exposure *in vitro*; 55.6 mg/kg intraperitoneally every 2 days *in vivo*) increased tumor lysis by 13–14% at 6 hours, boosted TNF-α/IFN-γ secretion, expanded stem-like and central-memory subsets 2.1- to 3.3-fold, reduced PD-1^+^TIM-3^+^ exhausted cells by half, and upregulated ECM-remodeling genes (MMPs, collagens) that enhanced tumor infiltration; *in vivo*, butyrate co-administration significantly improved tumor regression and survival in both xenograft models (15).

In CD19-directed CAR-T cells generated from healthy-donor T cells and tested against Raji lymphoma cells, sodium butyrate exposure (dose-dependent, 0.06–1 mM; sustained for 12 days) increased CD69/CD25 activation, specific tumor lysis, IL2/GZMB expression, and the proportion of transduced CAR^+^ cells, while shifting differentiation toward a CD8-skewed, less senescent (reduced CD27^−^CD28^−^CD57^+^, KLRG1, β-galactosidase), Th9-polarized phenotype, with 155 differentially expressed genes on RNA-seq; orally administered butyrate (150 mmol/L in drinking water) significantly prolonged survival in Raji-bearing NSG mice receiving human CD19 CAR-T cells (P = .025) (23).

In a ROR1-directed CAR-T model, murine CD8^+^ ROR1-CAR T cells pretreated with butyrate (0.75 mM, 3 days) or pentanoate showed increased CD25 expression and TNF-α/IFN-γ production; pentanoate-pretreated CAR-T cells (the metabolite carried forward into the *in vivo* experiments) significantly reduced tumor volume and weight and increased intratumoral IFN-γ^+^TNF-α^+^ CAR-T frequency in a PancROR1 xenograft model, with comparable activation effects of butyrate confirmed in human ROR1-CAR T cells against K562-ROR1 targets (35).

Because SCFAs exert pleiotropic and context-dependent effects across immune-cell subsets, controlled *ex vivo* conditioning may represent a more predictable translational strategy than systemic modulation, although this approach remains experimental (1, 18).

Evidence for valerate is less extensive than for butyrate, although direct preclinical and exploratory clinical CAR-T data are available.

In a large cohort of CD19 CAR-T recipients with large B-cell lymphoma (US discovery cohort, n = 67; German validation cohort, n = 58), higher pre-infusion stool valeric acid levels, measured endogenously by mass spectrometry, trended toward improved PFS in both cohorts (P = .071 and P = .081, respectively), although this association did not reach statistical significance; broad-spectrum antibiotics targeting anaerobic commensals (piperacillin-tazobactam/carbapenems) significantly depleted stool valeric acid alongside other SCFAs (18).

Direct functional evidence comes from *ex vivo* conditioning experiments. In human CD19-directed CAR-T cells (against NALM6 target cells), exposure to a range of SCFA concentrations (10–500 μM) enhanced cytotoxicity specifically in long-term serial-killing assays with repeated antigen exposure, without affecting single-round (24-hour) killing (18). When human CD19 CAR-T cells were cultured with 1 mM valeric acid for 7 days and then sorted into CD4^+^ and CD8^+^ fractions, untargeted metabolomics revealed a distinct metabolic profile characterized by increased acetylcarnitine, carnitine, deoxycarnitine, and several acylcarnitines (propionylcarnitine, C4- and C6-carnitine), indicating enhanced fatty acid oxidation, together with increased purine and pyrimidine nucleotide levels consistent with greater biosynthetic capacity for cell expansion; this metabolic reprogramming was not linked to a clinical or *in vivo* antitumor endpoint in this study (18). Succinate has been associated with increased spare respiratory capacity (34), whereas valerate-induced accumulation of carnitine and acylcarnitine intermediates is consistent with enhanced fatty-acid oxidation (18). Because memory-like CAR-T-cell states preferentially rely on mitochondrial oxidative phosphorylation and fatty-acid utilization relative to highly differentiated effector states (9), these metabolic effects provide a plausible link between microbiota-associated metabolites, CAR-T-cell persistence, and functional fitness.

In a murine ROR1-directed CAR-T model, CD8^+^ ROR1-CAR T cells pretreated with pentanoate (2.5 mM, 3 days) showed increased CD25 expression and TNF-α/IFN-γ production upon stimulation; *in vivo*, pentanoate-pretreated ROR1-CAR T cells significantly reduced tumor volume and weight and increased intratumoral IFN-γ^+^TNF-α^+^ CAR-T frequency in a PancROR1 (ROR1-expressing Panc02) xenograft model. The same effect was reproduced in human ROR1-CAR T cells: pentanoate pretreatment (dose-dependent, up to 7 mM, 4 days) enhanced CD25 expression, IL-2/IFN-γ/TNF-α secretion, proliferation, and cytolytic activity against ROR1-expressing K562 target cells *in vitro* (35). However, in this same study, systemic *in vivo* administration of pentanoate (oral or via anti-PD-1 combination) did not enhance antitumor immunity, in contrast to the *ex vivo* pretreatment approach (35).

Collectively, direct CAR-T studies support the capacity of selected SCFAs, particularly butyrate and, to a lesser extent, valerate, to enhance metabolic fitness and favor memory-associated differentiation, whereas the magnitude and mechanisms of these effects remain metabolite-, dose-, exposure duration-, and context-dependent — with *ex vivo* conditioning during manufacturing consistently outperforming systemic *in vivo* administration across studies.

From a metabolic-fitness perspective, these findings suggest that selected microbiota-associated metabolites may support CAR-T-cell states characterized by greater reliance on mitochondrial oxidative phosphorylation (OXPHOS) and fatty-acid oxidation, metabolic features commonly associated with less differentiated and more persistent memory-like T-cell phenotypes. Succinate-associated increases in spare respiratory capacity and valerate-associated enrichment of carnitine and acylcarnitine intermediates are consistent with enhanced mitochondrial respiratory flexibility and fatty-acid utilization. However, whether these metabolic changes are sufficient to causally improve long-term CAR-T persistence and antitumor efficacy remains to be established across products and disease settings.

### 6.3 Receptor signaling: indoles, kynurenine, and inosine

Microbiota- and tumor-derived metabolites can also regulate CAR-T-cell function through receptor-mediated pathways. Tryptophan metabolism is particularly relevant because it generates metabolites capable of engaging the aryl hydrocarbon receptor (AhR), although the functional consequences of AhR signaling depend on the cellular and metabolic context. Microbiota-derived indoles can activate AhR and modulate T-cell differentiation and function (7). In a recent preclinical study, Marcos-Kovandzic and colleagues used a fully immunocompetent syngeneic mouse model of B-cell lymphoma (C57BL/6J mice bearing a λ-MYC–driven, CD19-expressing B lymphoma, following lymphodepleting conditioning with 4 Gy total-body irradiation) treated with murine CD19/CD28-ζ CAR-T cells. Daily oral gavage of live *Akkermansia massiliensis* (strain p2261, ~10^9^ bacteria/day) alongside CAR-T infusion promoted the production of tryptophan-derived indole metabolites and enhanced CAR-T-cell cytotoxic (Tc1) polarization, with a lower intratumoral CD4/CD8 ratio, increased bone-marrow CAR-T-cell infiltration, and improved tumor control and overall survival compared with CAR-T cells alone. Consistent findings were observed in a parallel patient cohort receiving anti-CD19 CAR-T therapy for B-cell lymphoma, in whom baseline intestinal *Akkermansia* positivity was associated with higher 6-month overall response rate and longer progression-free survival. Importantly, the antitumor benefit of *Akkermansia* supplementation *in vivo* was lost when CAR-T cells were genetically deficient in AhR, indicating that AhR engagement was required for the *Akkermansia*-mediated benefit (33). However, individual indoles, including indole-3-propionic acid administered alone (by oral gavage *in vivo* or added to CAR-T/tumor cocultures *in vitro*), were insufficient to reproduce the effect of the live bacterium, suggesting that multiple microbial metabolites acting in combination, or additional *Akkermansia*-dependent mechanisms, are involved.

AhR signaling should therefore not be considered uniformly beneficial: studies in conventional tumor-infiltrating T cells have shown that specific tryptophan-derived metabolites can instead drive dysfunctional or exhausted phenotypes through AhR activation (1).

This context dependency is further illustrated by the tumor-derived indoleamine 2,3-dioxygenase 1 (IDO1)–kynurenine pathway. In a colorectal cancer model, ARID5A overexpression or CRISPR/Cas9 knockout in HCT116 and HT29 tumor cell lines was used to modulate IDO1 mRNA stability, tested against both HER2-specific and CEA-specific CAR-T cells *in vitro* (72-hour co-culture) and in NSG xenograft mice. ARID5A-driven IDO1 upregulation increased tumor-derived kynurenine production (measured by HPLC) and promoted nuclear AhR translocation in CD8^+^ CAR-T cells, resulting in upregulation of exhaustion markers (PD-1, TIM-3, LAG-3), reduced IFN-γ/TNF-α secretion, and impaired cytotoxicity against tumor cells; this exhausted phenotype was reproduced *in vivo*, with ARID5A-overexpressing tumors showing accelerated growth and reduced CAR-T-mediated tumor control. Genetic knockout of IDO1 in ARID5A-high tumors, or pharmacological AhR inhibition with SR-1 (1 μM), reversed this exhaustion phenotype and restored cytotoxic function, both *in vitro* and *in vivo* (reduced tumor volume, improved survival), while tryptophan deprivation similarly blocked AhR nuclear entry and exhaustion. Findings were corroborated in patient-derived colorectal cancer organoids and clinical samples, where high ARID5A/kynurenine levels correlated with CAR-T exhaustion markers (36). Thus, microbiota-derived indoles and tumor-derived kynurenine can engage the same AhR receptor while producing markedly different functional consequences in CAR-T cells — a beneficial, cytotoxicity-promoting effect in the Akkermansia/indole setting versus an exhaustion-driving effect in the IDO1/kynurenine setting.

A complementary pathway involves inosine. Previous studies in conventional T cells showed that microbiota-derived inosine, including that produced by Bifidobacterium pseudolongum, can signal through the adenosine A2A receptor (A2AR) and promote type 1 immune responses (14).

More recently, direct CAR-T evidence has emerged from a prospective cohort of 47 patients with relapsed/refractory diffuse large B-cell lymphoma undergoing CD19-directed CAR-T therapy (tisagenlecleucel or anbal-cel), in whom pretreatment stool and serum were profiled by whole-genome shotgun metagenomics and metabolomics (16). Gut *Bacteroides fragilis* abundance and functional enrichment of microbial inosine-biosynthesis pathways were associated with clinical response, and pretreatment serum inosine correlated significantly with *Bacteroides fragilis* abundance (R² = 0.355, P <.001) and was higher in responders than non-responders (P = .042); however, submedian serum inosine remained an independent negative predictor of progression-free survival on multivariate Cox regression (HR 2.78, 95% CI 1.14–7.53, P = .024), together with age and elevated ferritin. Higher serum inosine levels in responders and the independent association between submedian inosine and inferior PFS were directionally consistent. However, the supraphysiological inosine concentrations required to enhance CAR-T-cell cytotoxicity *in vitro* complicate the translational interpretation of these findings and leave the concentration–response relationship and the functional consequences of A2AR engagement at physiologically relevant inosine levels unresolved. In parallel *in vitro* experiments, healthy-donor-derived CD19 CAR-T cells cocultured with Nalm6 target cells showed enhanced cytotoxicity following inosine exposure (10–30 mM) both as a co-treatment and as a 48-hour pretreatment; this enhancement was attenuated by pharmacological A2AR inhibition, supporting an A2AR-dependent mechanism. No murine or other *in vivo* CAR-T model was used in this study; the antitumor benefit of inosine was demonstrated only *in vitro*, while its association with survival was based on an observational clinical cohort. Furthermore, serum inosine cannot be attributed exclusively to microbial production, because circulating levels may also reflect host purine metabolism, and the authors themselves note that these findings are exploratory and hypothesis-generating, requiring prospective validation in larger, multiethnic cohorts.

Together, these findings position inosine as a promising microbiota-associated metabolite linked to CAR-T efficacy, supported by mechanistic *in vitro* data and an A2AR-dependent mode of action, but not yet by direct *in vivo* reproduction of the antitumor effect.

### 6.4 Beyond SCFAs: amino acids, bile acids, polyamines, and lactate

Additional amino acid–dependent pathways can influence CAR-T-cell expansion and function, although their relationship with the gut microbiota remains less directly established than for SCFAs or indole metabolites. Arginine availability is particularly relevant in nutrient-restricted tumor environments: low extracellular arginine impairs CAR-T-cell proliferation, whereas engineering CAR-T cells to express the arginine-resynthesis enzymes argininosuccinate synthase (ASS) or ornithine transcarbamylase (OTC) enhances their proliferation under arginine-limited conditions and improves antitumor activity *in vivo* (37). Methionine provides an additional example of metabolic competition within the tumor microenvironment, although current mechanistic evidence derives predominantly from conventional CD8+ T cells. Tumor-mediated methionine restriction decreases intracellular S-adenosylmethionine and H3K79 dimethylation, thereby impairing T-cell survival and effector function (38). In contrast, direct CAR-T evidence supports a role for branched-chain amino acid (BCAA) metabolism: BCAA supplementation enhances CAR-T-cell proliferation and cytotoxicity, while metabolic reprogramming through BCKDK overexpression promotes antitumor activity, increased circulating CAR-T-cell abundance, and a central-memory phenotype in preclinical models (39). Collectively, these findings highlight amino acid availability and utilization as additional determinants of CAR-T-cell metabolic fitness, while their specific contribution to microbiota-mediated modulation of CAR-T therapy remains to be established.

Microbiota-dependent bile acid metabolism represents an additional, albeit indirect, pathway through which the gut microbiome may shape the environment encountered by CAR-T cells. Microbiota-transformed bile acids signal through receptors including the farnesoid X receptor (FXR), Takeda G protein-coupled receptor 5 (TGR5), and vitamin D receptor (VDR), thereby modulating macrophages, dendritic cells, NK cells, myeloid-derived suppressor cells, and conventional T-cell populations within the tumor immune microenvironment (40). Direct evidence linking these bile acid pathways to CAR-T-cell function, however, remains limited.

Polyamine metabolism may have more direct relevance to engineered T-cell fitness. In preclinical CAR-T models, spermidine pretreatment enhanced antigen-dependent proliferation, favored a central-memory-like phenotype, increased IL-2 and IFN-γ production, and improved CAR-T-cell cytotoxicity *in vitro* (41). Spermidine-pretreated nanobody-based CAR-T cells also showed evidence of improved antitumor activity and prolonged survival in lymphoma-bearing mice, although the *in vivo* benefit requires further validation.

Unlike several microbiota-derived metabolites discussed above, tumor-derived lactate is primarily considered a component of the metabolically hostile tumor microenvironment, although its biological effects are context dependent. High lactate concentrations, particularly when coupled with hypoxia and extracellular acidosis, can suppress T-cell proliferation, glycolytic activity, cytokine production, and cytolytic function, while also favoring immunosuppressive populations such as regulatory T cells and M2-like macrophages (42). Although much of this evidence derives from conventional and adoptively transferred T-cell models rather than CAR-T cells specifically, these conditions are highly relevant to CAR-T therapy because engineered T cells must function within the same acidic, lactate-rich tumor microenvironment.

### 6.5 Functional convergence and the metabolic network

Current evidence suggests that microbiota-associated modulation of CAR-T efficacy is unlikely to be attributable to a single metabolite. Rather, distinct microbial metabolites converge on interconnected processes relevant to CAR-T-cell fitness, including cellular metabolism, mitochondrial function, epigenetic regulation, receptor-mediated signaling, differentiation, and resistance to dysfunction.

Clinical metabolomic studies support this systems-level view. Broad-spectrum antibiotic exposure in CAR-T recipients is associated with simultaneous alterations across multiple classes of microbiota-associated metabolites, including short-chain fatty acids, other anionic metabolites, indole and cresol derivatives, and trimethylamine N-oxide, together with poorer clinical outcomes (18). These findings suggest that antibiotic-induced dysbiosis disrupts microbial metabolic output at a network level rather than affecting a single pathway. Consistent with this concept, individual indole metabolites failed to reproduce the CAR-T-enhancing effects of *Akkermansia* culture supernatant or live bacterial supplementation, suggesting that combinations of microbial metabolites or additional bacterium-dependent signals may be required for the full biological effect (33).

The gut microbiota can be viewed as a distributed metabolic system shaping CAR-T-cell fitness. This may explain the limited reproducibility of taxonomic signatures across cohorts and supports pathway- and metabolite-based approaches as more functionally informative than taxonomy alone.

## 7 Beyond systemic immunity: microbiota-mediated modulation of the tumor microenvironment

Microbiota-derived metabolites may influence CAR-T efficacy not only through direct effects on engineered T cells but also indirectly by shaping the immunological and metabolic environment in which these cells operate. However, the nature and magnitude of tumor-microenvironmental constraints differ substantially across CAR-T indications. Importantly, although several mechanisms of microbiota-mediated tumor-microenvironment (TME) remodeling have been demonstrated in general tumor biology and checkpoint-blockade models, direct evidence that the microbiota modifies CAR-T efficacy specifically through TME remodeling remains limited. These distinctions are therefore essential when interpreting the relevance of hypoxia, lactate accumulation, adenosine signaling, myeloid suppression, and stromal remodeling across different CAR-T products.

### 7.1 CD19-directed CAR-T in lymphoid malignancies

In CD19-directed CAR-T therapy, malignant cells are predominantly located in blood, bone marrow, and lymphoid tissues rather than within the dense, poorly perfused stromal architecture characteristic of many solid tumors. Accordingly, although hypoxia, lactate-driven extracellular acidosis, and adenosine signaling may also contribute to immunosuppression in B-cell malignancies, metabolic constraints and, particularly, physical stromal barriers are well-established obstacles to CAR-T efficacy in solid tumors (43). Nevertheless, metabolically restrictive conditions may occur within lymph-node or bone-marrow niches, and myeloid cells remain highly relevant to CAR-T biology, particularly as mediators of systemic inflammation and treatment-related toxicities such as CRS and ICANS (31, 32). Direct clinical evidence in CD19 CAR-T recipients supports associations between antibiotic exposure, microbiota disruption, microbial composition, metabolite profiles, and clinical outcomes (16–19, 23). However, these studies do not establish that microbiota-derived metabolites influence CD19 CAR-T efficacy specifically through remodeling of local lymphoid or marrow microenvironments. At present, direct effects on CAR-T-cell metabolic fitness and systemic host–microbiota interactions therefore remain better supported than a microbiota-driven TME mechanism.

### 7.2 BCMA-directed CAR-T in multiple myeloma

BCMA-directed CAR-T cells encounter the distinct metabolic and immunological environment of the bone marrow. The myeloma marrow is characterized by relative hypoxia, metabolic competition, stromal-cell interactions, suppressive myeloid populations, and immunoregulatory signaling, all of which may constrain T-cell function (44). Importantly, direct microbiota–CAR-T evidence has emerged in this setting. In patients receiving BCMA-directed CAR-T therapy, microbiota-associated metabolic features, including pre-infusion stool succinate concentrations, have been associated with CAR-T-cell persistence, while experimental succinate supplementation enhanced central-memory differentiation and respiratory capacity in CAR-T cells (34). These findings establish a link between microbiota-associated metabolic output and BCMA CAR-T biology but do not demonstrate that the effect is mediated through remodeling of the bone-marrow microenvironment. Whether microbiota-associated metabolites act predominantly through local marrow remodeling, systemic metabolic exposure, or direct cell-intrinsic effects on CAR-T cells therefore remains unresolved.

### 7.3 Solid-tumor CAR-T

Solid tumors represent the setting in which metabolic and structural TME barriers are most pronounced. Hypoxia, nutrient deprivation, extracellular acidosis, lactate accumulation, adenosine signaling, suppressive myeloid-cell infiltration, and dense stromal architecture can collectively impair CAR-T-cell trafficking, expansion, mitochondrial function, cytokine production, cytotoxicity, and persistence (45). Unlike hematologic malignancies, physical exclusion by extracellular matrix and tumor-associated stroma represents an additional barrier that can limit CAR-T-cell penetration into tumor tissue. Myeloid-derived suppressor cells and tumor-associated macrophages further reinforce local immunosuppression through metabolic competition and inhibitory signaling. Although these mechanisms are increasingly recognized as major determinants of solid-tumor CAR-T failure, direct evidence that the intestinal microbiota modulates these specific barriers during CAR-T therapy remains scarce.

Most mechanistic evidence supporting microbiota-mediated TME remodeling in solid tumors currently derives from non-CAR-T experimental systems. In pancreatic cancer models, gut-derived lipopolysaccharide reached tumor tissue and increased tumor-cell PD-L1 expression through TLR4/MyD88/AKT/NF-κB signaling while altering intratumoral T-cell infiltration and exhaustion (30). Conversely, in colorectal cancer models, *Lactobacillus gallinarum*-derived indole-3-carboxylic acid reduced tumor IDO1 expression and kynurenine production, attenuated kynurenine-dependent AhR signaling, decreased regulatory T-cell infiltration, and enhanced CD8+ T-cell effector activity (46). These mechanisms were demonstrated in general tumor-immunity or checkpoint-blockade settings rather than in CAR-T models. They therefore establish biological plausibility for microbiota-mediated remodeling of the solid-tumor microenvironment but should not be interpreted as direct evidence of microbiota-dependent modulation of solid-tumor CAR-T therapy.

Taken together, the relationship between the microbiota, the TME, and CAR-T-cell function is strongly context dependent. Direct microbiota–CAR-T evidence is currently concentrated in hematologic malignancies, where clinical and translational studies primarily support associations with systemic metabolic output, CAR-T-cell phenotype, persistence, response, and toxicity. By contrast, the biological rationale for microbiota-mediated TME remodeling is particularly compelling in solid tumors, where hypoxia, lactate and adenosine accumulation, myeloid suppression, and stromal exclusion represent major therapeutic barriers, but the microbiota-dependent component of these mechanisms remains largely extrapolated from non-CAR-T tumor-immunity models. Future studies should therefore avoid generalizing TME mechanisms across CAR-T products and instead determine, within the relevant anatomical compartment, whether microbiota-derived signals modify nodal or marrow niches in CD19- and BCMA-directed therapies or the metabolically and structurally restrictive stroma encountered by solid-tumor CAR-T cells (47, 48).

## 8 Therapeutic strategies for microbiota modulation: from evidence to intervention

In light of the proposed metabolome-centered framework, the therapeutic goal is not simply to restore microbial composition, but to preserve or reconstitute its metabolic output.

### 8.1 FMT, targeted supplementation, and diet

Fecal microbiota transplantation (FMT) represents the most comprehensive strategy for restoring both microbial diversity and metabolic function by simultaneously transferring an entire microbial ecosystem. In cancer immunotherapy, responder-derived FMT combined with immune checkpoint blockade has been associated with reversal of treatment resistance in a subset of patients with programmed cell death protein 1 (PD-1)-refractory melanoma, achieving response rates of approximately 30% in early-phase studies (6). In hematologic malignancies, available evidence supports the biological rationale and feasibility of FMT, although efficacy data in CAR-T recipients remain limited and are currently under investigation (49). A randomized trial evaluating FMT in lymphoma patients receiving CAR-T therapy (NCT06218602) is ongoing (18).

Despite its promise, FMT faces important practical limitations, including infectious risks in profoundly immunocompromised patients, potential transmission of multidrug-resistant organisms, and the need for rigorous donor screening and standardized protocols (24, 49). Evidence from hematologic and broader microbiome studies suggests that the biological effects of microbiota restoration may depend on recovery of ecosystem-level properties, including microbial diversity, mucosal homeostasis, and functional metabolic output, rather than on the presence of individual taxa alone. These functions include the production of SCFAs, tryptophan-derived metabolites, bile-acid derivatives, and other immunomodulatory molecules, although their specific contribution to FMT efficacy in CAR-T recipients has not yet been established.

Alternative microbiome-modulating approaches aim to restore or support microbial metabolic function without transferring an entire microbial ecosystem. These strategies include targeted microbial consortia, prebiotics, postbiotics, and metabolite-based interventions, although their efficacy in CAR-T recipients remains largely investigational. Likewise, probiotic supplementation should not be applied indiscriminately in neutropenic or severely immunocompromised patients, as its safety and potential biological effects are highly dependent on the microbial strain, preparation, and clinical context (1, 3, 24).

Dietary interventions represent a complementary microbiome-modulating strategy. Evidence from broader cancer immunotherapy settings suggests that increased intake of fermentable dietary fiber may support SCFA production and has been associated with improved clinical outcomes in selected cohorts, although direct evidence in CAR-T recipients remains limited (6). The effectiveness of such interventions is likely to depend on the presence of a sufficiently preserved fermentative microbial community; accordingly, their impact may be reduced in patients with profound treatment- or antibiotic-related dysbiosis in whom key commensal strains have been depleted (24, 49).

### 8.2 Protection of the microbiota during antibiotic exposure

Because exposure to selected broad-spectrum antibiotics, particularly regimens with substantial anti-anaerobic activity, has been associated with microbiome and metabolome disruption and inferior outcomes in several CAR-T cohorts, preservation of microbiome function during antimicrobial treatment represents a rational complementary strategy, although these clinical associations remain observational and potentially confounded (18).

When clinically feasible, antibiotic stewardship approaches, including avoidance of unnecessary broad-spectrum exposure and timely de-escalation, may help limit collateral disruption of microbial diversity and metabolic function, although their impact on CAR-T efficacy has not yet been established prospectively (18, 24). Beyond stewardship, several emerging approaches are being investigated to reduce collateral microbiome damage while maintaining antimicrobial efficacy.

Gut-protective enzymatic approaches, including orally administered β-lactamases, have been shown outside the CAR-T setting to reduce intestinal exposure to selected systemically administered β-lactam antibiotics and to limit antibiotic-associated microbiome disruption. Their use in CAR-T recipients has not yet been evaluated (3). Because these agents act within the gastrointestinal tract while preserving systemic antimicrobial activity, they could represent a microbiome-sparing strategy in CAR-T recipients, although this remains hypothetical. Similarly, preclinical studies suggest that strain-specific bacteriophages can selectively deplete targeted gut bacteria with greater precision than broad-spectrum antibiotics. Whether such approaches could be used to eliminate pathobionts while preserving beneficial metabolite-producing commensals in CAR-T recipients remains to be determined (7, 14).

Overall, microbiota protection should be viewed not as an alternative to appropriate antimicrobial therapy, but as an effort to preserve the metabolic integrity of the intestinal ecosystem during periods of unavoidable antibiotic exposure.

### 8.3 *Ex vivo* metabolic conditioning of CAR-T cells

Metabolic conditioning during CAR-T manufacturing offers a unique opportunity to directly modulate the cellular product, potentially reducing, at least in part, the influence of interindividual variability in microbiota-derived metabolic signals at the time of infusion. However, the manufacturing process does not begin from a biologically neutral substrate. Autologous leukapheresis-derived T cells are collected from patients whose prior therapies, disease burden, infections, systemic inflammatory state, and immunosuppressive exposures may already have altered T-cell composition and fitness. Clinical and translational evidence indicates that these factors can affect naïve and memory T-cell representation, proliferative capacity, differentiation state, and susceptibility to exhaustion, thereby influencing both manufacturing performance and the functional quality of the final CAR-T product (50–52).

An intriguing but largely unexplored possibility is that the gut microbiota may influence CAR-T manufacturing before the manufacturing process begins by shaping the biological state of leukapheresis-derived T cells. In this hypothetical framework, antibiotic-induced dysbiosis and reduced availability of microbiota-derived metabolites could contribute to a circulating T-cell pool with impaired metabolic fitness or increased susceptibility to dysfunction. However, direct evidence linking microbiota composition or metabolite availability to leukapheresis T-cell quality or subsequent CAR-T manufacturing outcomes is currently lacking. This hypothesis is nevertheless biologically plausible given the established relationship between CAR-T immunometabolic fitness, memory-associated differentiation, persistence, and therapeutic efficacy (9, 10).

Within this framework, microbiota-derived metabolites may serve as potential manufacturing tools rather than solely as systemic interventions. Evidence from conventional T-cell biology indicates that SCFAs can modulate epigenetic and metabolic pathways involved in T-cell differentiation and function, whereas the extent to which specific mechanisms such as HDAC inhibition and chromatin remodeling operate in CAR-T cells remains incompletely defined. Direct CAR-T studies nevertheless support functional effects of SCFA exposure: butyrate promotes a central-memory phenotype, reduces senescence-associated features, enhances cytotoxicity, and improves antitumor activity in preclinical models (23), while *ex vivo* exposure to SCFAs, including valerate, enhances CAR-T metabolic fitness and sustained tumor-killing capacity during repeated antigen stimulation (18).

Importantly, metabolite-based conditioning should be considered as a biomanufacturing variable rather than simply as a culture supplement. Studies of cytokines, metabolic modulators, signaling-pathway inhibitors, and epigenetic agents indicate that their effects on CAR-T-cell phenotype and function can depend on concentration, timing, duration of exposure, and integration with the activation and expansion protocol (53). Accordingly, key unresolved questions include whether metabolite conditioning is most effective during early activation or later expansion, whether induced metabolic and phenotypic changes persist after cryopreservation and infusion, and whether such approaches can be incorporated into standardized, GMP-compatible manufacturing workflows.

Overall, *ex vivo* metabolic conditioning represents a promising translational opportunity emerging from microbiota–CAR-T research. By directly modulating CAR-T-cell metabolic fitness during manufacturing, this strategy offers a comparatively controllable intervention that could reduce, at least in part, dependence on interpatient variability in microbiota-derived metabolic signals while providing a direct experimental route for translating microbiome-derived discoveries into cellular manufacturing strategies (**Supplementary Table S2**).

The integration of microbiota- and metabolome-related influences across the CAR-T treatment trajectory, from the pre-leukapheresis state through manufacturing, infusion, and tumor microenvironment exposure, together with the potential positioning of *ex vivo* metabolic conditioning, is illustrated in **Figure 3**.

**Figure 3:**
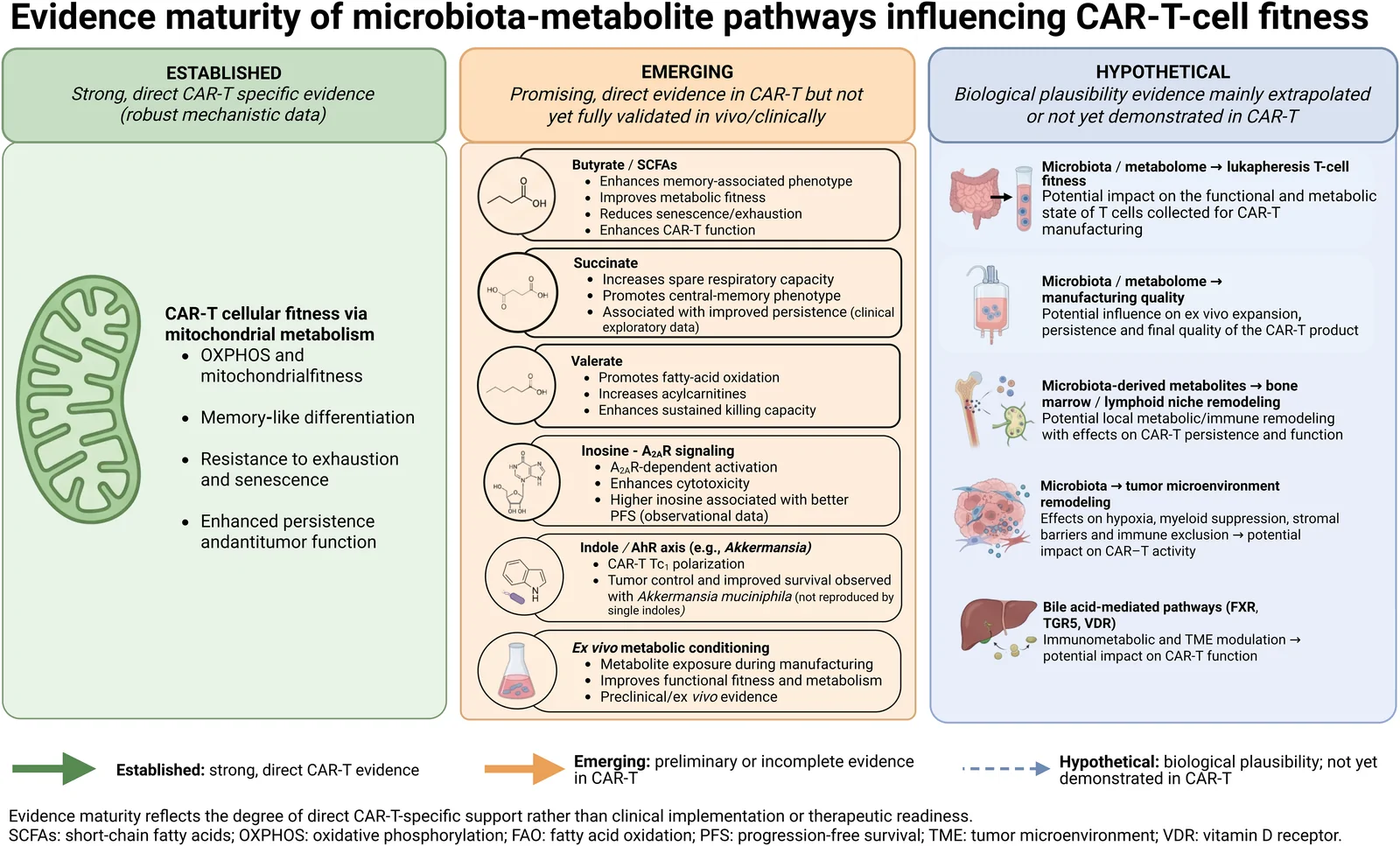
Evidence maturity of microbiota–metabolite pathways influencing CAR-T-cell fitness. Pathways are classified as established, emerging, or hypothetical according to the strength of CAR-T-specific evidence.

### 8.4 Toward an integrated strategy

Collectively, these approaches illustrate the evolution of the field from descriptive microbiome studies toward increasingly interventional strategies. Importantly, current evidence suggests that therapeutic benefit may depend less on restoration of individual bacterial taxa than on preservation or reconstruction of broader ecosystem functions, including microbial metabolic output, although this concept remains to be prospectively validated in CAR-T recipients.

Current strategies can be viewed along a continuum of intervention specificity. FMT, dietary modulation, and targeted microbial supplementation seek to reshape the intestinal ecosystem itself, whereas microbiota-protective approaches aim to limit disruption of its functional output during treatment. *Ex vivo* metabolic conditioning represents a distinct strategy in which microbiome-derived insights are applied directly during CAR-T-cell manufacturing, thereby potentially reducing dependence on the patient’s microbiota at the time of infusion.

Although most of these interventions remain investigational, emerging clinical and preclinical evidence supports a conceptual framework in which microbiota-derived metabolites may contribute to CAR-T-cell fitness, persistence, and antitumor activity. Importantly, the strength of this evidence varies substantially across metabolites and intervention types, ranging from observational clinical associations to direct mechanistic studies in CAR-T models. Future multidisciplinary studies integrating microbiome profiling, metabolomics, and manufacturing-focused investigations will be essential to determine which of these strategies can be translated into clinically actionable interventions.

Among these emerging approaches, *ex vivo* metabolic conditioning is particularly attractive because it enables direct and experimentally controllable manipulation of the CAR-T product; however, its clinical value, optimal implementation, and superiority over alternative microbiota-targeted strategies remain to be established.

## 9 Toward precision metabolomics: an integrated predictive framework

Current evidence indicates that no single biological layer is sufficient to accurately predict CAR-T outcomes.

As discussed above, the framework proposed here integrates microbial metabolic output across the full CAR-T trajectory, including the manufacturing process — an axis that has received limited attention to date.

Microbiome sequencing identifies microbial composition and metabolic potential but cannot determine the functional state of the ecosystem. Conversely, metabolomics provides a dynamic readout of microbial and host biological activity but must be interpreted within the broader context of immune status, CAR-T-cell characteristics, prior therapies, antibiotic exposure, inflammation, and clinical variables.

These considerations support the development of an integrated systems-level framework centered on precision metabolomics, host immune characterization, CAR-T-cell features, and clinical variables. Such integration is particularly relevant because CAR-T therapy is inherently dynamic. Static baseline measurements are unlikely to capture the dynamic biological changes occurring from leukapheresis through manufacturing, infusion, and post-treatment follow-up.

Longitudinal evaluation of microbial composition, circulating and fecal metabolites, immune activation, cytokine profiles, and CAR-T-cell phenotypes may help identify dynamic signatures associated with expansion, toxicity, durable response, or early relapse (16–18, 21). Among potential time points, metabolomic profiling at the time of infusion may be especially informative because it reflects the cumulative impact of prior therapies, antibiotic exposure, dysbiosis, inflammation, and residual immune fitness at the moment CAR-T cells encounter the host environment.

The ultimate objective is to define patient-specific metabolic–immunological fingerprints capable of complementing existing clinical predictors and guiding personalized interventions, including antibiotic stewardship, microbiota restoration, dietary modulation, metabolite-based therapies, and *ex vivo* metabolic conditioning. Although still exploratory, precision metabolomics provides a promising translational framework for integrating microbiota-derived functional information into predictive models and next-generation CAR-T optimization.

## 10 Open questions and future directions

Despite rapid progress, several key questions must be addressed before microbiota- and metabolome-based strategies can be integrated into routine CAR-T practice.

First, future studies should identify which metabolites, or combinations of metabolites, have sufficient mechanistic and preclinical support to justify interventional trials. Although SCFAs, indoles, inosine, succinate, bile acids, amino-acid-derived metabolites, and polyamines have all emerged as candidates, their translational maturity varies substantially.

Second, the optimal timing and compartment for profiling remain unclear. Whether stool metabolomics, circulating metabolomics, or integrated multi-omics approaches provide the most actionable information, and at which stages of the CAR-T treatment trajectory, requires prospective evaluation.

The possibility that the microbiota influences CAR-T therapy before infusion, and potentially even before manufacturing begins, deserves dedicated investigation. Future studies should determine whether microbiome and metabolomic profiles correlate with leukapheresis quality, T-cell fitness, manufacturing efficiency, and final product potency.

Finally, predictive signatures and intervention strategies must be validated across diverse geographic, dietary, clinical, and technological settings. The field should therefore move toward multicenter longitudinal studies designed not only to predict outcomes but also to identify clinically actionable interventions.

## 11 Conclusions

The gut microbiota has emerged as an important extratumoral determinant of CAR-T-cell therapy outcomes. The major conceptual advance in this field is no longer the identification of microbial taxa associated with response, but the recognition that microbiota-derived metabolic functions may represent the biological layer most directly linked to CAR-T-cell efficacy, persistence, and toxicity.

From this perspective, microorganisms are not the final signal but components of a coordinated metabolic network acting on CAR-T biology. Loss of immunomodulatory metabolites, including SCFAs, indoles, and inosine, represents one plausible mechanism through which dysbiosis and antibiotic exposure may impair therapeutic responses, whereas preservation of microbial metabolic output is associated with more favorable immunometabolic states.

Accordingly, the field is shifting from a descriptive framework centered on microbial composition to a functional and translational model in which the metabolome emerges both as a biomarker platform and as a therapeutic target. Within this framework, antibiotic stewardship, microbiota restoration, dietary modulation, metabolite-based interventions, and *ex vivo* metabolic conditioning should be viewed not as competing strategies but as complementary approaches aimed at preserving or reconstructing beneficial metabolic programs.

Important limitations remain. Much of the available clinical evidence is observational, transferability of microbial signatures across cohorts is incomplete, and prospective validation of biomarkers and interventions is still ongoing. Ultimately, optimizing CAR-T therapy may require engineering not only the cellular product itself but also the metabolic ecosystem in which it is generated and ultimately functions.
