# Supplementary material for: Microbiota-derived metabolic priming of CAR-T cell fitness: a translational framework for next-generation cell therapy
Source: Front Immunol. 2026 Sep 22;17:1953443. doi: 10.3389/fimmu.2026.1953443 (PMC13639960; doi:10.3389/fimmu.2026.1953443)
Supplement: Supplementary file 1 [file Table1.docx]

**Table S1. Experimental and mechanistic evidence for metabolic pathways relevant to CAR-T-cell fitness**

| **Metabolite** | **Experimental model / CAR target** | **Exposure (route & concentration/dose)** | **Principal endpoint** | **Dose-dependent / opposing effects** | **Evidence level & translational potential** |
| --- | --- | --- | --- | --- | --- |
| **Butyrate (SCFA)** (15,23,35) | Human MSLN-CAR-T, xenograft (15); human CD19 CAR-T vs Raji (23); murine/human ROR1-CAR-T (35) | 2 mM, 2 d + 55.6 mg/kg IP q2d in vivo (15); 0.06–1 mM, 12 d + 150 mmol/L drinking water in vivo (23); 0.75 mM, 3 d (35) | ↑ memory phenotype, ↓ exhaustion, ↑ cytotoxicity; improved tumour regression/survival in vivo (15,23) | Class I HDAC inhibition shared with valerate/pentanoate; supraphysiological blockade could in principle impair CAR-T proliferation/activation (narrow window documented in other T-cell contexts), not directly tested here | Preclinical; ex vivo conditioning |
| **Valerate (SCFA)** (18) | Human CD19 CAR-T recipients, LBCL, discovery/validation cohorts ; human CD19 CAR-T vs NALM6, ex vivo | Endogenous stool valerate, clinical; 10–500 µM / 1 mM, 7 d ex vivo | Trend toward improved PFS, not significant; ↑ serial-killing cytotoxicity | Enhanced long-term serial-killing capacity, whereas no improvement was observed in single-round 24-h cytotoxicity; no direct in vivo antitumor validation reported | Clinical + in vitro preclinical; biomarker and ex vivo conditioning potential |
| **Succinate** (34) | Human BCMA-CAR-T (ARI0002h), r/r myeloma, n=18 evaluable; murine FOS-diet xenograft | Pre-infusion stool succinate, endogenous; 20 mmol/L, final 3 d of expansion | Persistence correlated with stool succinate (R=0.444, P=.044); ↑ central-memory, ↑ respiratory capacity; enhanced persistence/tumour control in vivo, OS unchanged | Exposure- and context-dependent; 20 mmol/L during CAR-T expansion promoted a memory-associated phenotype and respiratory capacity, while the optimal concentration–response relationship remains unresolved. | Clinical + preclinical; metabolic supplementation |
| **Inosine** (16) | Human CD19 CAR-T recipients, r/r DLBCL, n=47; healthy-donor CD19 CAR-T vs Nalm6, in vitro | Serum inosine, endogenous; 10–30 mM (supraphysiological), co-treatment/48 h pretreatment | Higher serum inosine in responders (P=.042); submedian inosine an independent negative predictor of PFS (HR 2.78, P=.024); ↑ cytotoxicity in vitro, A2AR-dependent — not reproduced in vivo | Higher inosine was associated with favorable outcomes, but the supraphysiological concentrations required in vitro leave the physiologically relevant concentration–response relationship and A2AR effects unresolved. | Clinical + in vitro; biomarker candidate |
| **Indoles metabolites / IPA** (33) | Syngeneic murine CD19/CD28-ζ CAR-T lymphoma model; parallel human anti-CD19 CAR-T cohort | *A. massiliensis* p2261, 1 × 10⁹ bacteria/day by oral gavage; IPA, 100 µL/day by oral gavage (concentration not reported); 10% *Akkermansia* supernatant ex vivo | ↑ BM CAR-T infiltration, Tc1 polarization and tumour control/OS with *Akkermansia*; benefit abolished in AhR-deficient CAR-T cells; IPA alone ineffective in vivo | AhR-dependent benefit requires the broader *Akkermansia*-derived metabolic context; individual indoles/IPA alone do not reproduce the effect | Clinical association + preclinical mechanistic; microbiota/AhR modulation |
| **Kynurenine** (36) | HER2- and CEA-specific CAR-T vs CRC models; 72 h coculture; NSG xenografts; patient-derived organoids | Tumour-derived via ARID5A-driven IDO1 upregulation; AhR inhibitor SR-1, 1 µM | ↑ PD-1/TIM-3/LAG-3, ↓ IFN-γ/TNF-α and cytotoxicity; impaired tumour control in vivo; reversed by IDO1 knockout or AhR inhibition | Same AhR receptor as microbiota-derived indoles but opposite functional outcome; AhR signalling is context-dependent | Preclinical + translational; IDO1/AhR inhibition |

*SCFA, short-chain fatty acid; HDAC, histone deacetylase; AhR, aryl hydrocarbon receptor; A2AR, adenosine A2A receptor; IPA, indole-3-propionic acid; IDO1, indoleamine 2,3-dioxygenase 1; TME, tumour microenvironment; PFS, progression-free survival; OS, overall survival; LBCL, large B-cell lymphoma; DLBCL, diffuse large B-cell lymphoma; MSLN, mesothelin; ↑, increase/higher; ↓, decrease/lower.*
