# Supplementary material for: Microbiota-derived metabolic priming of CAR-T cell fitness: a translational framework for next-generation cell therapy
Source: Front Immunol. 2026 Sep 22;17:1953443. doi: 10.3389/fimmu.2026.1953443 (PMC13639960; doi:10.3389/fimmu.2026.1953443)
Supplement: Supplementary file 2 [file Table2.docx]

**Table S2. Representative ex vivo conditions reported in the literature for CAR-T/T-cell manufacturing modulation**

| **Molecule** | **Concentration** | **Manufacturing phase** | **Biological effect** | **Main limitation** |
| --- | --- | --- | --- | --- |
| Butyrate (C4:0 SCFA) [35] | 0.75 mM (Luu; CTL/ROR1 CAR-T) | Expansion/activation (3 days post-activation) | Class I HDAC inhibition enhances CD8+ effector-memory function and antitumour activity | Effects demonstrated primarily in murine models; human CAR-T validation limited to short-term functional readouts (cytokine secretion, proliferation, cytotoxicity) without long-term persistence or in vivo human data |
| Valerate / Pentanoate (C5:0 SCFA) [35] | 2.5 mM (murine CTL/ROR1 CAR-T); 4 mM (human CTL/CAR-T) | Expansion/activation (3-4 days post-activation) | Class I HDAC inhibition enhances mTOR-associated CTL/CAR-T activation, cytotoxicity, and antitumour efficacy. | Limited availability, higher dosing needs, weak CAR-T memory evidence, and unclear GMP applicability. |
| Spermidine (polyamine) [41] | 5 µM main dose; beneficial range 0.5-5 µM; higher doses showed dose-dependent viability impairment in the T-cell assay | Expansion (4 days after CAR-T generation/transduction) | Central-memory enrichment improves CAR-T proliferation, effector function, exhaustion profile, and in vivo tumour control. | Narrow window with limited CAR-T evidence, modest in vivo benefit, and unclear translational feasibility. |
| Succinate (TCA intermediate) | - | - | - | No direct ex vivo CAR-T conditioning data in the selected literature. |
| Methionine (essential AA) | - | - | - | No direct ex vivo CAR-T conditioning data in the selected literature. |

*AA, amino acid; CAR-T, chimeric antigen receptor T-cell; CTL, cytotoxic T lymphocyte; GMP, good manufacturing practice; HDAC, histone deacetylase; mTOR, mechanistic target of rapamycin; ROR1, receptor tyrosine kinase-like orphan receptor 1; SCFA, short-chain fatty acid; TCA, tricarboxylic acid.*
